# Integrated Metabolomic and Transcriptomic Analyses Reveal the Basis for Carotenoid Biosynthesis in Sweet Potato (*Ipomoea batatas* (L.) Lam.) Storage Roots

**DOI:** 10.3390/metabo12111010

**Published:** 2022-10-23

**Authors:** Qingming Ren, Xiaoxi Zhen, Huiyu Gao, Yinpei Liang, Hongying Li, Juan Zhao, Meiqiang Yin, Yuanhuai Han, Bin Zhang

**Affiliations:** 1 College of Agriculture, Shanxi Agricultural University, Jinzhong 030801, China; 2 Shanxi Key Laboratory of Minor Crops Germplasm Innovation and Molecular Breeding, Shanxi Agricultural University, Jinzhong 030801, China; 3 Ministerial and Provincial Co-Innovation Centre for Endemic Crops Production with High-Quality and Efficiency in Loess Plateau, Shanxi Agricultural University, Jinzhong 030801, China

**Keywords:** sweet potato, carotenoid, abscisic acid, metabolome, transcriptome

## Abstract

Carotenoids are important compounds of quality and coloration within sweet potato storage roots, but the mechanisms that govern the accumulation of these carotenoids remain poorly understood. In this study, metabolomic and transcriptomic analyses of carotenoids were performed using young storage roots (S2) and old storage roots (S4) from white-fleshed (variety S19) and yellow-fleshed (variety BS) sweet potato types. S19 storage roots exhibited significantly lower total carotenoid levels relative to BS storage roots, and different numbers of carotenoid types were detected in the BS-S2, BS-S4, S19-S2, and S19-S4 samples. β-cryptoxanthin was identified as a potential key driver of differences in root coloration between the S19 and BS types. Combined transcriptomic and metabolomic analyses revealed significant co-annotation of the carotenoid and abscisic acid (ABA) metabolic pathways, *PSY* (phytoene synthase), *CHYB* (β-carotene 3-hydroxylase), *ZEP* (zeaxanthin epoxidase), *NCED3* (9-cis-epoxycarotenoid dioxygenase 3), *ABA2* (xanthoxin dehydrogenase), and *CYP707A* (abscisic acid 8’-hydroxylase) genes were found to be closely associated with carotenoid and ABA content in these sweet potato storage roots. The expression patterns of the transcription factors OFP and FAR1 were associated with the ABA content in these two sweet potato types. Together, these results provide a valuable foundation for understanding the mechanisms governing carotenoid biosynthesis in storage roots, and offer a theoretical basis for sweet potato breeding and management.

## 1. Introduction

Sweet potato (*Ipomoea batatas* (L.) Lam.) is an annual worldwide member of the Convolvulaceae family, representing an important food source and cash crop throughout the world [1]. Sweet potato storage roots contain high levels of carbohydrates, minerals, dietary fiber, and bioactive ingredients [2]. Yellow-fleshed sweet potato types are rich in β-carotene and other provitamin A carotenoids [3]. The color of sweet potato storage roots is considered highly important, in part because it can inform consumer choices [4]. There is thus a clear need to fully explore the interplay between carotenoid biosynthesis and storage root color development in order to better guide the selective breeding of sweet potatoes with a high level of commercial value.

Carotenoids are isoprenoid-derived pigments that are vital for the growth of the plants [5]. In addition to determining the coloration of the flesh of fruits and roots in which they accumulate, these carotenoids can exert a range of antioxidant and antitumor activities in humans, with some studies suggesting that these bioactive compounds can prevent a variety of ocular diseases [6,7]. The pigmentation of sweet potato storage roots is primarily determined by the apparent carotenoid and anthocyanin discrepancy [8,9]. While orange- and yellow-fleshed storage roots contain similar carotenoids, the actual differing levels of particular carotenoids within the roots of different sweet potato types have been extensively studied in many plant species. [10,11,12].

Carotenoid biosynthesis has been studied at length in plants including *Arabidopsis* [13], tomatoes [14], peppers [15], rice [16], and maize [17]. This process is governed by a series of controlled reactions including condensation [14], dehydrogenation [18], cyclization [19], hydroxylation [20], and epoxidation [17]. The phytoene synthase (PSY)-mediated condensation of 20-carbon geranylgeranyl diphosphate (GGPP) molecules to generate colorless phytoene (C40) represents a critical step in this biosynthetic process [21]. Lycopene is then generated through successive phytoene desaturation and isomerization. Phytoene desaturase (PDS), z-carotene isomerase (Z-ISO), z-carotene desaturase (ZDS), and carotenoid isomerase (CRTISO) catalyze four different dehydrogenation reactions [22]. During the process of carotenoid cyclization reactions, competition between lycopene beta cyclase (LCY-β) and lycopene epsilon cyclase (LCY-ε) governs the relative production of β-carotene and α-carotene [12], with the double hydroxylation of these two respective products yielding lutein and zeaxanthin, after which further modification of zeaxanthin can yield neoxanthin and violaxanthin [17]. These latter two carotenoids serve as precursors for the biosynthesis of the phytohormone abscisic acid (ABA), which relies on the enzymatic cleavage of 9-cis-violaxanthin or 9′-cis-neoxanthin (9-cis-epoxy-xanthophylls), mediated by NCED, to produce C15-xanthoxin and C25-apocarotenoid [23]. After transfer from plastids into the cytoplasm, xanthoxin then undergoes two further processing steps to yield ABA, whereas apocarotenoid can be converted by ABA2 (short-chain dehydrogenase/reductase-like enzyme ABA-deficient 2), after which it can be oxidated by AAO (ABA by abscisic aldehyde oxidase) to yield ABA [24]. Further metabolic processing of ABA can then be performed by enzymes, including ABA-8′-hydroxylases and CYP enzymes of the 707A clade, producing major catabolites, including phaseic acid (PA) and dihydrophaseic acid (DPA) [25].

Carotenoid production is primarily shaped by the expression of genes that regulate the different steps in these biosynthetic pathways [26], with differential gene expression ultimately accounting for the observed differences in carotenoid content among species and types. In yellow celery, lutein and β-carotene content is closely associated with AgLCYB and AgPSY2 expression levels [20]. Analyses of mutant Oranzheva kaoia types, exhibiting high concentrations of β-carotene, have revealed the presence of a biosynthetic pathway breakdown owing to CrtZchr03 gene deletion, ultimately contributing to increased β-carotene content and affirming the regulatory role of particular genes in this metabolic context [27]. Studies of mutant Cara cara navel oranges have further demonstrated that the DXS1, DXR, GGPPS2, PSY1, and LCYB genes are the primary determinants of carotenoid biosynthesis [9].

Comprehensive multi-omics analyses have been widely employed to study the mechanisms whereby plant pigmentation is established. In *Cyclocarya paliurus*, for example, the MYB transcription factors (TFs) and two bHLH TFs were identified via a multi-omics approach as important regulators of flavonoid biosynthesis [28]. Similarly, integrative metabolomic and transcriptomic analyses have enabled the determination of the mechanisms that regulate anthocyanin and flavonoid accumulation within sweet potato root skin and leaf vein base tissues [29]. By leveraging these multi-omics techniques, researchers have also established a putative transcriptional regulatory network that dictates flavonoid and carotenoid biosynthesis in navel oranges, enabling the development of a hierarchical model for proposed pathway-related genes and TFs [9]. Similar strategies have also supported studies concerning the relationship between carotenoid biosynthesis and petal color in *Brassica napus* [26].

To date, most studies of sweet potato carotenoids have primarily focused on phenotypic characteristics or derivation rather than on underlying metabolic and molecular processes. In the present study, the storage roots of the white-fleshed S19 and the yellow-fleshed BS sweet potato types were selected for systematic metabolomic and transcriptomic analyses aimed at exploring the mechanisms underpinning carotenoid accumulation in these sweet potato storage roots. Ultimately, this approach highlighted key metabolites and genes associated with the coloration of these sweet potato storage roots while offering novel mechanistic insight into the basis for sweet potato carotenoid biosynthesis.

## 2. Materials and Methods

### 2.1. Plant Materials and Treatments

The white-fleshed, red-skinned S19 variety (*Ipomoea batatas* (L.) Lam. cv ‘Shangshu 19’) and the yellow-fleshed, light yellow-skinned BS variety (*Ipomoea batatas* (L.) Lam. cv ‘Baishu’) were selected for experimental use.

On 30 May 2020, these two sweet potato types were planted on the experimental agricultural farm of Shanxi Agricultural University (coordinates: 112.58′ E, 37.42′ N). They were planted in a random block arrangement, with two rows of 50 plants per variety using a single ridge planting approach with 35 cm between plants, and a row spacing of 90 cm. Sample collection was performed on day 90 (S1), day 100 (S2), day 110 (S3), and day 120 (S4) after planting. In total, five plants per variety were harvested at random for each time point, yielding 20 storage roots. Similarly sized storage roots with smooth skin were selected for subsequent characterization.

Harvested sweet potato roots were rinsed, dried with absorbent paper, and cut into 0.5 cm × 0.5 cm × 0.5 cm cubes that were snap-frozen for 30 min in liquid nitrogen and stored at −80 °C.

### 2.2. Analyses of Total Carotenoid Content and Color

A colorimetric approach was used to assess the average carotenoid content in BS and S19 sweet potato storage root samples. The color was assessed using a X Rite VS450 colorimeter (Xrite, Grand Rapids, MI, USA). Carotenoid content following crude organic solvent-mediated extraction was assessed as per Lambert–Beer’s law as follows:

(1) A = α CL

where A corresponds to the absorbance of a given solution, C corresponds to the concentration of the reactant, L corresponds to liquid layer thickness, and α corresponds to the absorption coefficient.

Each sample was analyzed in triplicate using biological replicates.

### 2.3. Quantification of Carotenoid Content and ABA Levels

After freeze-drying, samples of detached sweet potato storage roots from the BS and S19 types at the S2 and S4 time points were ground using a mixer mill. An UPLC-APCI-MS/MS system (UPLC, ExionLC^TM^ AD, https://sciex.com.cn/ (accessed on 4 November 2020); MS Applied Biosystems 6500 Triple Quadrupole, https://sciex.com.cn/ (accessed on 4 November 2020)) and the MetWare (http://www.metware.cn/ (accessed on 4 November 2020)) application were then used to assess the carotenoid and phytohormone content in these samples, with all analyses having been performed by the MetWare company. Principal component analysis (PCA) and orthogonal partial least squares discriminant analysis (OPLS-DA) approaches were used to evaluate differences in metabolite profiles among these samples. Differentially abundant metabolites (DAMs) were identified using defined significance criteria: variable importance in projection ((VIP) ≥ 1, |log2 FC (fold change)| ≥ 1, and *p* < 0.05 (Student’s *t*-test)). The Kyoto Encyclopedia of Genes and Genomes (KEGG) database was then used to map DAMs and to assess significant enrichment thereof, and to define key enriched pathways.

### 2.4. qPCR

Prior to the experimental operation, the bench was treated with a Solid RNase scavenger (Coolaber, Beijing, China). Frozen sweet potato storage root samples were ground in liquid nitrogen using a mortar and pestle to produce a powder from which total RNA was extracted using RNaiso Plus (Takara Biotechnology, Beijing, China). An ultra-low-volume spectrometer (BioDrop, Cambridge, UK) was utilized to measure the concentration and A260/A280 ratios for these samples, with cDNA then being prepared with a PrimeScript™ RT reagent Kit with gDNA Eraser (Takara Biotechnology, Beijing, China), based on provided directions for RNA samples with an A260/A280 of 1.8–2.1. Prior to qPCR analyses, these cDNA samples were subject to 5-fold dilution. A Bio-Rad CFX96 Real-Time PCR instrument and TB Green^®^ Premix Ex Taq™ II (Takara Biotechnology, Dalian, China) were used for qPCR with the following settings: 95 °C for 30 s; 40 cycles of 95 °C for 5 s; 60 °C for 30 s. A melting curve from 60 °C to 95 °C (0.5 °C increments for 5 s) was used. The ∆∆Cq approach was used to assess relative gene expression [30] in the Bio-Rad Manager 3.1 software, with Actin serving as a control to normalize gene expression levels. Primer Premier 5.0 was used to design all primers for this study (Table S8).

### 2.5. RNA-Sequencing

For RNA-Seq analyses, Oligo(dT) magnetic beads were used to isolate polyadenylated mRNA, while the removal of rRNA from total RNA samples was additionally used to isolate mRNA. A splitting buffer was used to break RNA molecules into shorter strands that then served as templates for first-strand cDNA synthesis using random hexamer primers. Then, dNTPs (dUTP, dATP, dGTP, dCTP), DNA polymerase I, and first-strand cDNA were used for second-strand cDNA synthesis, followed by the use of AMPure XP beads to isolate prepared cDNA. Following the addition of a poly-A tail, samples were then concatenated using a sequencing adapter, and a final cDNA library was generated through PCR-based enrichment. A Qubit 2.0 instrument was used to quantify cDNA library size, with insert size being assessed using an Agilent 2100 instrument, and qPCR being used to measure the effective concentration. After cDNA library quality had been confirmed, an Illumina Hi-Seq instrument was used for sequencing.

### 2.6. Quality Control and Bioinformatics Analyses

After sequencing, cleaned reads were obtained through filtering, error rate analyses, and assessments of GC content in the obtained raw reads. The Trinity software was used to derive reference sequences, and clustering was performed with the Corset (https://code.google.com/p/corset-project/ (accessed on 10 November 2020)) tool, which enables the establishment of gene-level counts based on de novo transcriptomic assemblies in which the most extended cluster sequences are designated as a unigene. MetWare (http://www.metware.cn/ (accessed on 4 November 2020)) performed all analyses. Unigenes were compared with the KEGG and the Gene Ontology (GO) databases using the BLAST software, and predicted amino acid sequences for protein-coding unigenes were established, after which they were compared with the protein family (Pfam) database using the HMMER program. Differentially expressed genes (DEGs) were compared between samples using DESeq2 in R, with Benjamini–Hochberg correction being used to control for the *p*-value false discovery rate (FDR) when performing multiple comparisons. DEGs were ultimately identified based on the following criteria: |log2FC| ≥ 1 and FDR < 0.05. The prediction of gene function was performed using cluster analyses, and distribution frequencies were assessed in all functional categories.

### 2.7. Statistical Analysis

SPSS 22.0 was used for statistical analyses of included samples. Data were compared using the Tukey test for multiple comparisons. Differences in physicochemical indices were compared using a minimum of three biological replicates for all experiments. Microsoft Office Excel 2016 was used for data analyses.

## 3. Results

### 3.1. Carotenoid Accumulation in Storage Roots Varies in Different Sweet Potato Types

**Figure 1:**
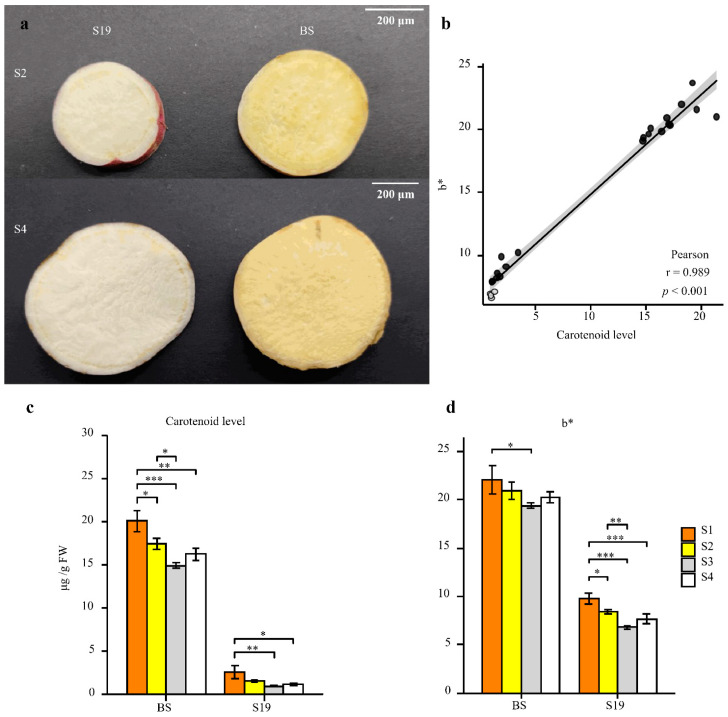
Analyses of sweet potato storage root phenotypic characteristics and carotenoid content. (**a**) Storage root phenotypes were assessed for BS-S2, BS-S4, S19-S2, and S19-S4 samples. Scale bar = 200 μm. (**b**) Correlations between carotenoid content and color change. (**c**,**d**) Total carotenoid content (**c**) and color change (**d**) were analyzed in both sweet potato types at the analyzed time points. Data are means ± SEM (*n* = 3). *** *p* < 0.001, ** *p* < 0.01, * *p* < 0.05.

**Figure 2:**
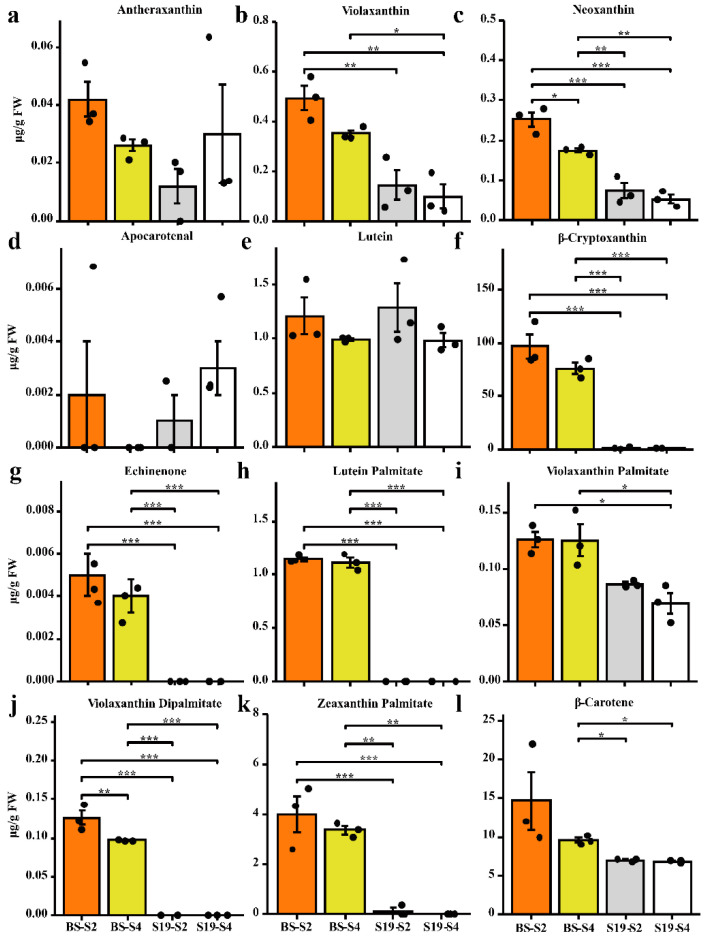
Analysis of carotenoid levels in sweet potato samples. The content of lutein (**a**–**g**), carotenoid esters (**h**–**k**), and carotene (**l**). Data are means ± SEM (*n* = 3). *** *p* < 0.001, ** *p* < 0.01, * *p* < 0.05.

During different stages of development, the coloration of S19 and BS storage roots were compared, revealing that these two types exhibited white and yellow storage root flesh, respectively (Figure 1a). In line with these phenotypes, BS samples contained 7-fold higher total carotenoid levels relative to S19 samples over the analyzed stages of development (S1–S4) (Figure 1c,d). Changes in color represented by the b * (yellow/white) value were significantly positively correlated with total carotenoid content (r^2^ > 0.95), and this trend remained consistent from stage S1 to stage S4 (Figure 1b–d). Carotenoid content in these storage root samples was then examined in further detail via LC-MS/MS, leading to the identification of 68 carotenoid types, including 14 types of lutein, 7 types of carotenes, and 47 types of carotenoid esters. The BS-S2, BS-S4, S19-S2, and S19-S4 samples contained 12, 12, 9, and 8 carotenoid types, respectively. Different trends in carotenoid content were observed in these two sweet potato types over the course of development. The most abundant carotenoid in the yellow-fleshed BS sweet potato storage roots was β-cryptoxanthin (81.41% in S2; 82.02% in S4), and these levels were 86- and 111-fold higher than the corresponding levels in white-fleshed S19 storage roots at the S2 and S4 stages, respectively (Figure 2f and Figure S1a,b). Whereas the most abundant carotenoid in S19 samples was β-carotene (69.18% in S2; 77.92% in S4), its total abundance remained significantly lower than that in BS storage roots during all stages of development (Figure 2l and Figure S1c,d). These results suggest that the contents of β-cryptoxanthin, β-carotene, and zeaxanthin palmitate may be the primary determinants of the overall differences in total carotenoid content observed in storage roots from these two types of sweet potato. Significant increases in violaxanthin, lutein palmitate, violaxanthin palmitate, violaxanthin dipalmitate, and echinenone content were also evident in BS storage roots relative to those from S19 sweet potatoes at the S2 and S4 stages of development, whereas no differences in antheraxanthin, apocarotenal, or lutein content were observed when comparing these two types. Principal component analysis (PCA) confirmed that there were significant differences between the metabolite profiles of these two sweet potato types, with PC1 and PC2 accounting for 61.9% and 15.7% of the variability among these samples, respectively (Figure S1e).

### 3.2. Changes in ABA Metabolism over the Course of Storage Root Development

**Figure 3:**
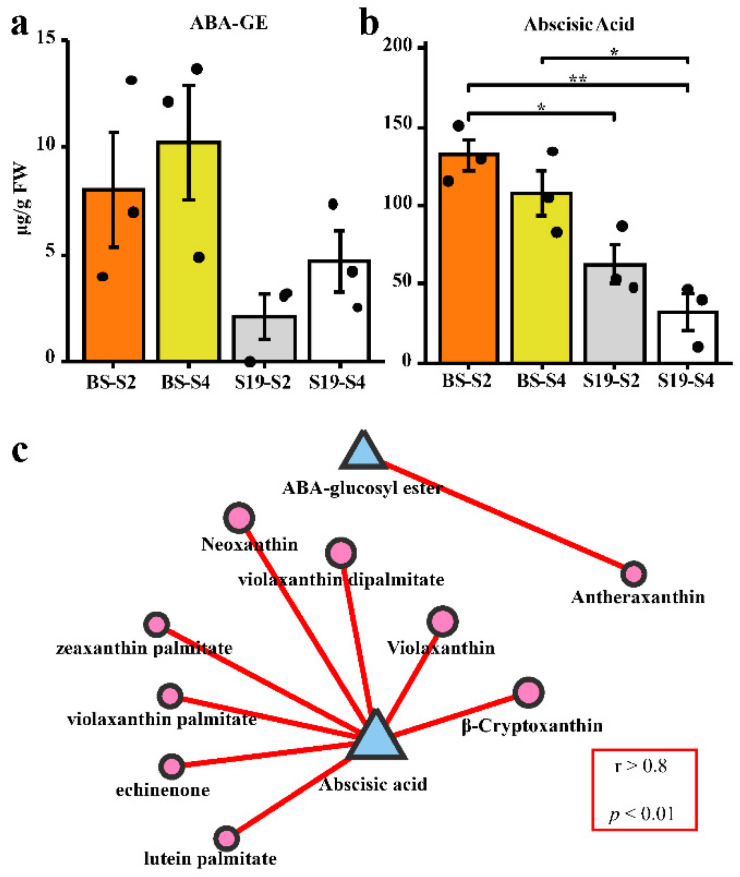
Correlations between ABA levels and carotenoid content. (**a**) Total ABA-GE levels in the indicated samples (μg/g FW). (**b**) Total ABA levels in the indicated samples (μg/g FW). (**c**) The contents of ABA and ABA-GE were positively correlated with the contents of carotenoid-related components (graphic area is positively correlated with content.). Data are means ± SEM (*n* = 3). ** *p* < 0.01, * *p* < 0.05.

ABA metabolism and its relationship with carotenoids in sweet potatoes were examined by using LC-MS/MS. Opposing trends in ABA and ABA-glucosyl ester (ABA-GE) content were observed in these storage roots, with ABA-GE levels trending upwards over the course of development in both sweet potato types, while ABA levels trended downwards. Total ABA content was significantly higher in BS storage roots than S19 storage roots (Figure 3a,b). As shown in Figure 3c, changes in the ABA content of these two sweet potato types were positively correlated with the observed trends in most carotenoid compositions. However, the change trend of ABA-GE content was only related to that of antheraxanthin content (Figure 3c).

### 3.3. Transcriptomic Analyses of BS and S19 Sweet Potato Storage Roots

To fully understand the molecular mechanisms that shape sweet potato carotenoid biosynthetic processes, a transcriptomic analysis of the S19 and BS sweet potato storage roots was conducted at the young (S2) and old (S4) stages of development. In total, analyses of these 12 samples yielded 79.17 GB of clean data (6 GB/sample), with Q30 base percentages of >90% and a GC content of 45% (Table S1). Over 84% of reads were mapped, including 70% uniquely mapped reads and 10% multiply mapped reads (Table S2).

**Figure 4:**
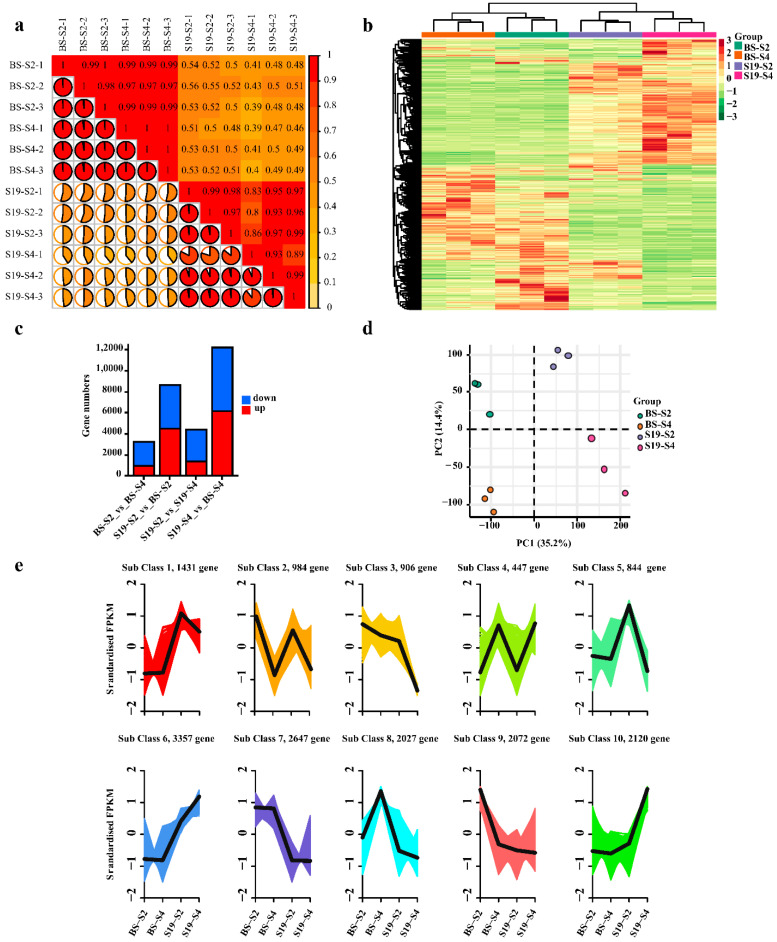
Analyses of DEGs identified in transcriptomic of sweet potato storage root samples and principal component analyses. (**a**) Sample repeatability and correlation analyses. (**b**) Heatmaps representing hierarchically clustered DEGs. (**c**) Statistical analyses of DEGs in the indicated groups. (**d**) PCA for samples included in transcriptomic analyses. (**e**) DEG cluster analyses.

DESeq2 was next used to identify differentially expressed genes (DEGs) in BS and S19 samples at these different stages of development (|log2FC| ≥ 1, FDR < 0.05). PCA and cluster analyses revealed close clustering of biological replicate samples, with clear distinctions between samples from different groups (Figure 4a–c). In total, these analyses identified 8643 (4470 upregulated and 4173 downregulated), 12,212 (6154 upregulated and 6058 downregulated), 3298 (983 upregulated and 2315 downregulated), and 4409 (1391 upregulated and 3018 downregulated) DEGs for the respective S19-S2_vs_BS-S2, S19-S4_vs_BS-S4, BS-S2_vs_BS-S4, and S19-S2_vs_S19-S4 comparisons (Figure 4d). In total, 10 subset classes were clustered based on DEG expression patterns (Figure 4e).

When the annotated DEGs in these groups were subject to GO classification based on the molecular function (MF), biological process (BP), and cellular component (CC) annotation categories (Table S3), 3800 DEGs (885 MF, 2491 BP, and 424 CC), 3883 DEGs (898 MF, 2547 BP, and 438 CC), 3064 DEGs (728 MF, 2048 BP, and 288 CC), and 3329 DEGs (728 MF, 2222 BP, and 325 CC) were annotated for these four respective comparisons (S19-S2_vs_BS-S2, S19-S4_vs_BS-S4, BS-S2_vs_BS-S4, and S19-S2_vs_S19-S4).

Specific metabolic pathways involved in the regulation of sweet potato storage root carotenoid biosynthesis in the BS and S19 were further explored through KEGG enrichment analyses for these four comparisons, revealing these DEGs to be significantly enriched in cellular processes, environmental information processing, genetic information processing, metabolism, and organismal systems. In total, 19, 11, 30, and 20 DEGs from the four respective comparisons (BS-S2_vs_BS-S4, S19-S2_vs_S19-S4, S19-S2_vs_BS-S2, and S19-S4_vs_BS-S4) were found to be significantly enriched in the carotenoid biosynthesis pathway (ko00906) (Figures S2–S4).

### 3.4. Combined Analyses of Metabolites and Genes Associated with Carotenoid Biosynthesis in Sweet Potato Storage Roots

**Figure 5:**
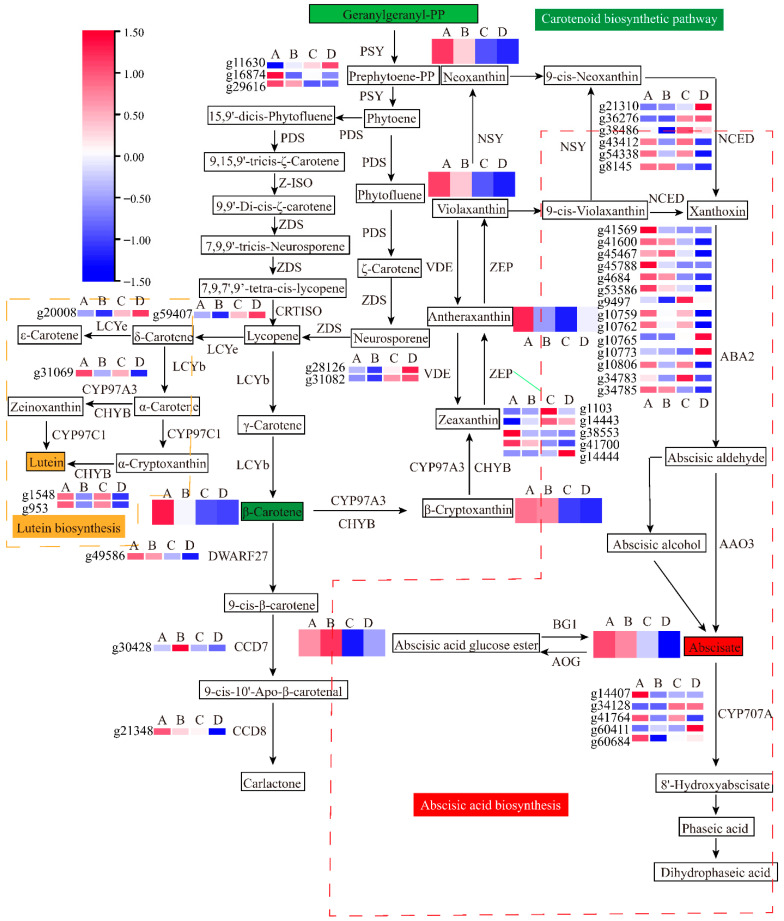
The carotenoid and ABA metabolic pathways exhibiting DEG and DAM enrichment. (A: BS-S2; B: BS-S4; C: S19-S2; D: S19-S4.).

To explore the metabolites and mechanisms associated with sweet potato storage root carotenoid accumulation, a combined transcriptomic and metabolomic analysis of BS and S19 samples during the different stages of development was conducted. In total, 43 DEGs and 7 differentially accumulated metabolites (DAMs) were associated with the carotenoid biosynthesis pathway (Tables S4 and S5), enabling the establishment of a metabolic profiling diagram for these carotenoid pathways (Figure 5). In total, 24 differentially expressed structural genes associated with the biosynthesis and catabolism of carotenoids were identified, including genes encoding PSY (15-cis-phytoene synthase), ZEBRA2 (prolycopene isomerase), ZEP (zeaxanthin epoxidase), VDE (violaxanthin de-epoxidase), NCED (9-cis-epoxycarotenoid dioxygenase), LCYE (lycopene epsilon-cyclase), and CHYB (β-carotene 3-hydroxylase).

**Figure 6:**
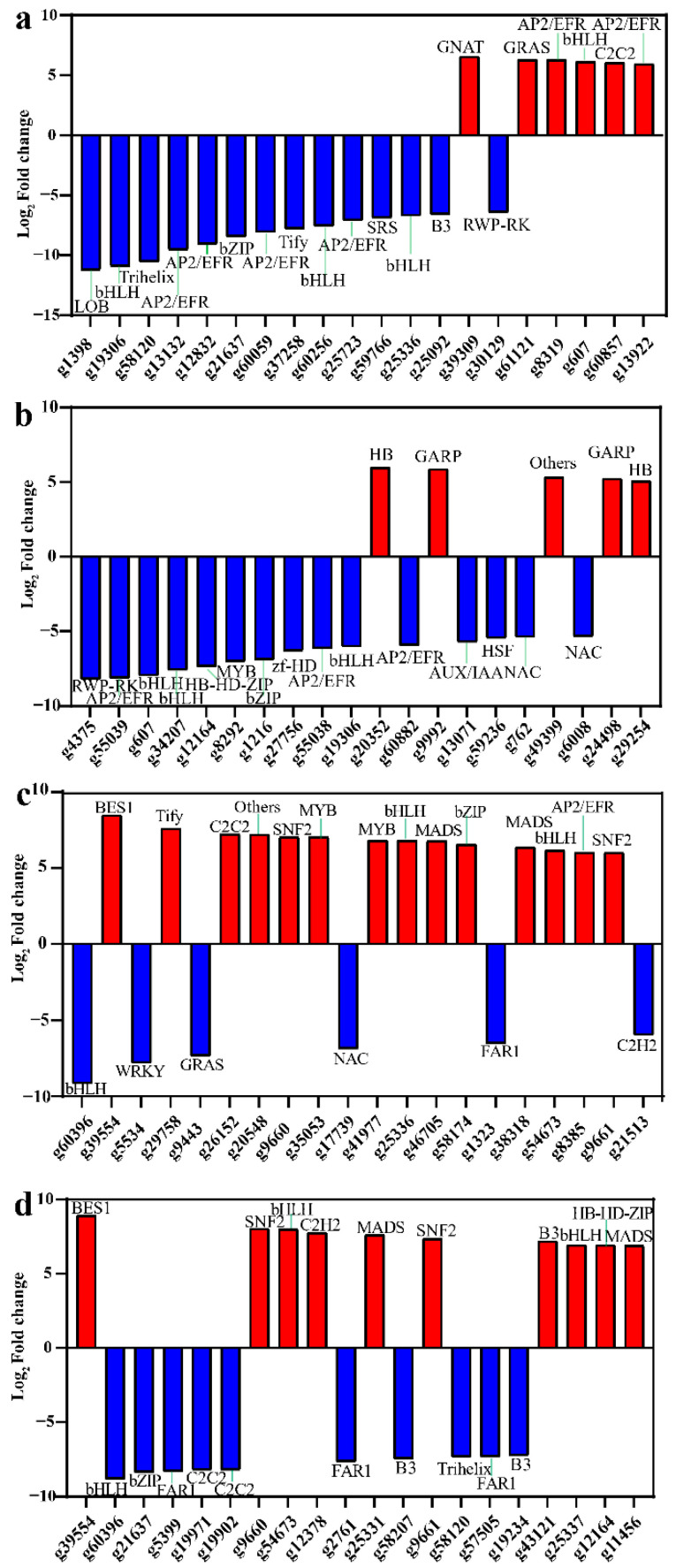
Differentially expressed genes encoding transcription factors in sweet potato samples. (**a**–**d**) The top 20 TF-encoding genes exhibiting the greatest fold-change in expression levels when comparing the BS-S2 and BS-S4 (**a**), S19-S2 and BS-S2 (**b**), S19-S2 and S19-S4 (**c**), and S19-S4 and BS-S4 (**d**) samples.

*PSY* (*g29616*) expression in BS has been shown to be significantly upregulated relative to levels in S19 storage roots at the S2 and S4 stages. *CHYB* expression in the storage roots of these sweet potato types trended downward from the S2 to the S4 stage, with *CHYB* gene (*g1548* and *g953*) expression levels in BS storage roots that were 3.56-, 3.85-, and 2.52-, 2.59-fold higher than those in the S19 storage root at the analyzed stages of development. Relative to BS storage roots, lower levels of *ZEP* (*g41700*) expression were evident in S19 storage roots, particularly during the S2 stage. However, *ZEP* genes (*g14444* and *g1103*) were expressed at very high levels in S19 samples, with opposing expression trends during different stages of development. *CCD* gene (*g49586*) expression was significantly increased in BS storage roots at the analyzed stages of development, as compared to those from S19 sweet potatoes. Relative *NCED3* (*g36276*) expression was increased by 34.09- and 39.74-fold in S19 storage roots during the respective S2 and S4 stages relative to levels in BS storage roots. Moreover, 8 ABA metabolism-associated DEGs were identified, including 14 ABA2 (xanthoxin dehydrogenase) and 5 CYP707A (abscisic acid 8’-hydroxylase). Downregulated expression of the *ABA2* gene was observed over the course of development, with significantly lower *ABA2* (*g34785*) expression in S19 storage roots relative to those from BS sweet potatoes. The ABA degradation-associated gene *CYP707A* (*g34128*) was expressed at 302.20- and 59.31-fold higher levels in S19 storage roots relative to BS storage roots at the S2 and S4 stages, respectively. Three key *ABA2* genes (*g4684*, *g34785*, and *g41600*) were also significantly positively correlated with ABA content (Figure 6). These DEGs may be associated with the observed differences in carotenoid metabolic activity in the BS and S19 types, and with differences in the levels of detected DAMS. β-carotene (C02094), β-cryptoxanthin (C08591), antheraxanthin (C08579), violaxanthin (C08614), neoxanthin (C13431), ABA (C06082), and ABA-GE (C15970) were detected (Figure 5, Table S5).

### 3.5. Identification of Transcription Factors Related to Carotenoid Accumulation

**Figure 7:**
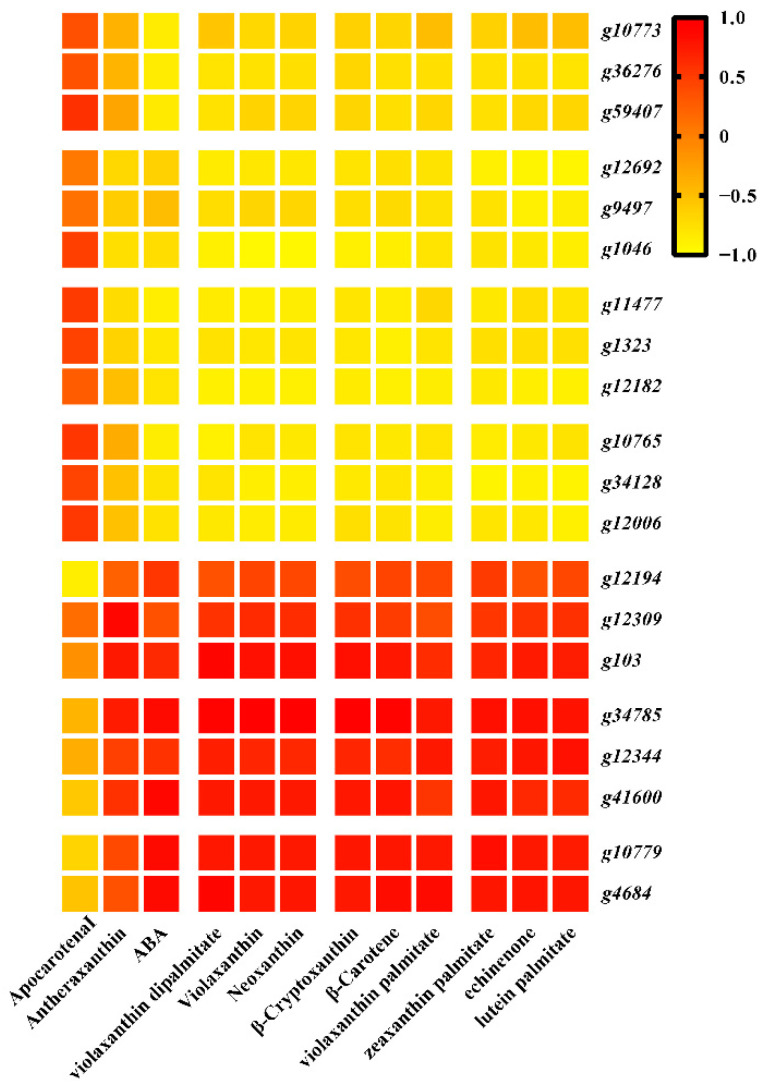
Correlation between DEGs and DAMs trends, with stronger correlations corresponding to a more consistent trend.

Transcription factors (TFs) and other transcriptional regulators (TRs) are key regulators of the expression of all genes, including those associated with the biosynthesis of carotenoids. In this study, 284 (88 upregulated, 196 downregulated), 255 (58 upregulated, 197 downregulated), 365 (231 upregulated, 134 downregulated), and 577 (335 upregulated, 242 downregulated) differentially expressed TF-coding genes were identified from the comparisons of BS-S2_vs_BS-S4, S19-S2_vs_S19-S4, S19-S2_vs_BS-S2, and S19-S4_vs_BS-S4, respectively. Moreover, these four respective comparisons yielded 44 (10 upregulated, 34 downregulated), 57 (18 upregulated, 39 downregulated), 89 (41 upregulated, 48 downregulated), and 126 (54 upregulated, 72 downregulated) differentially expressed TR-encoding genes (Table S6). These TFs included members of the AP2/ERF, MYB, bHLH, bZIP, NAC, FAR1, C2H2, PLATZ, WRKY, OFP, and TCP families, while identified TRs included members of the PHD, SET, SNF2, TAZ, and AUX/IAA, and SNF2 families (Table S7). Of these differentially expressed TFs and TRs, the most highly expressed included AP2/ERF, MYB, bHLH, AUX/IAA, SNF2, and PHD. When screening the top 20 DEGs encoding TFs, the genes associated with carotenoid biosynthesis were found to be upregulated when comparing S19 and BS storage roots, particularly during the S2 stage of development (Figure 7). The gene encoding NAC was expressed at higher levels in BS storage roots relative to S19 storage roots during both the S2 and S4 stages, although when comparing S2 vs. S4 samples, *NAC* (*g103*) was downregulated in BS storage roots yet upregulated in S19 storage roots. Strikingly, of the analyzed differentially expressed TF genes, *OFP* (*g10779*) and *FAR1* (*g1323*) expression levels differed significantly in the storage roots from the two sweet potato types, with *OFP* (*g10779*) being expressed at significantly higher levels in BS samples, whereas *FAR1* (*g1323*) was expressed at significantly higher levels in S19 samples at the S2 and S4 stages of development.

### 3.6. RNA-Seq Result Validation

**Figure 8:**
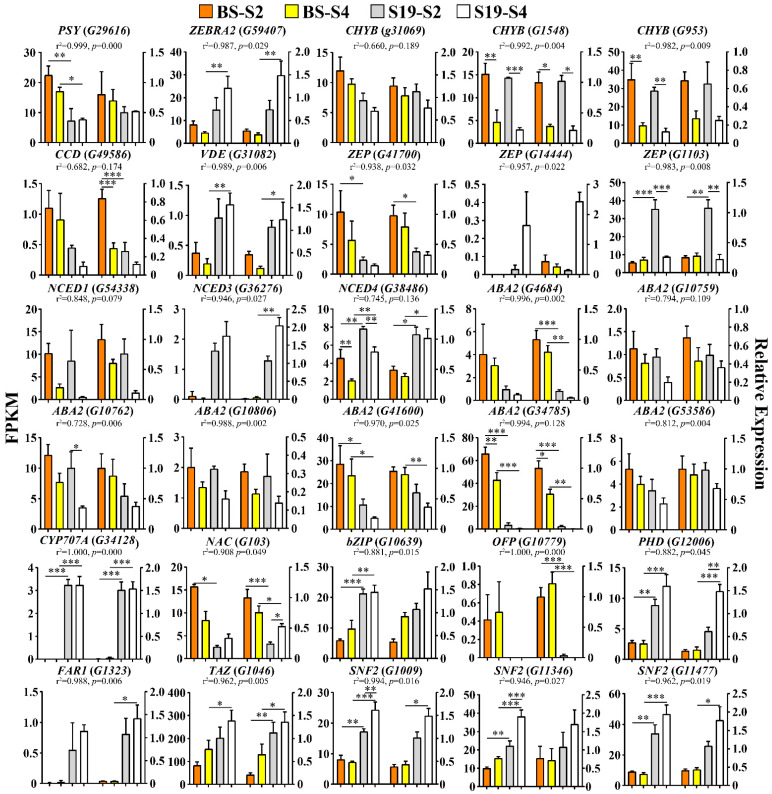
Expression analyses of structural genes and transcription factors associated with the carotenoid and ABA biosynthesis pathways. Data are means ± SEM (*n* = 3). *** *p* < 0.001, ** *p* < 0.01, * *p* < 0.05.

To confirm the validity of the RNA-Seq results established above, 13 DEGs associated with carotenoid biosynthesis, 8 DEGs associated with ABA biosynthesis, and 9 TFs closely associated with carotenoid biosynthesis were selected for qPCR-based verification analysis (Figure 8). Overall, the observed expression trends for these 30 genes were highly consistent with the RNA-Seq results presented above, confirming the accuracy and reliability of these transcriptomic sequencing analyses.

## 4. Discussion

In sweet potato storage roots, carotenoid content is a key determinant of coloration, appearance, and health-related benefits, thereby shaping consumer preference [31]. Indeed, the increase in total carotenoid content is strongly correlated with the changes in the color of these storage roots over the course of development. Sweet potato storage roots contain carotenoids such as β-carotene, β-cryptoxanthin, zeaxanthin, and violaxanthin [32]. Here, characterization of carotenoid content in storage root samples prepared from two sweet potato types at different stages of development revealed that BS storage roots contained significantly higher carotenoid levels relative to S19 storage roots, with marked differences in the levels of the two primary carotenoids in these storage roots (β-carotene and β-cryptoxanthin). The most abundant carotenoids in yellow-fleshed sweet potatoes included β-carotene and β-cryptoxanthin, with the latter being present at higher concentrations [10,11,12,32]. Consistently, β-cryptoxanthin was the most abundant carotenoid in yellow-fleshed BS storage roots, suggesting that it may be the primary determinant of the observed differences in coloration between these two sweet potato types.

Efforts to characterize carotenoid metabolism-related genes have been made in a range of species including tomatoes [14], carrots [33], oranges [34], and peppers [15]. The mechanisms shaping carotenoid biosynthesis in sweet potatoes, however, have yet to be firmly established. In an effort to identify key carotenoid accumulation-associated genes and metabolites, metabolomic and transcriptomic analyses were thus performed. PSY is a key rate-limiting carotenoid biosynthesis-associated enzyme in plants that can ultimately shape total carotenoid content [21,35]. Both RNA-Seq and qPCR analyses confirmed significantly increased *PSY* (*g29616*) expression in BS storage roots relative to those from S19 sweet potatoes during the tested developmental stages, potentially partially accounting for observed differences in carotenoid content in these two types. The CHYB enzyme is responsible for catalyzing the addition of a hydroxyl residue necessary for esterification, and is vital for chromoplast carotenoid accumulation in a range of plants. CHYB is a rate-limiting enzyme in the zeaxanthin biosynthesis pathway that has been shown to play a key role in chromoplast carotenoid accumulation in *Ipomoea petals* [36]. Consistently, BS storage roots exhibited higher *CHYB* (*g1548* and *g953*) expression levels relative to S19 storage roots, potentially contributing to differences in β-carotene and zeaxanthin content in BS and S19 samples. The ZEP enzyme catalyzes zeaxanthin and antheraxanthin β-rings epoxidation, and BS storage roots were herein found to express significantly higher levels of *ZEP* (*g41700*). In line with the present report, Suematsu et al. suggested that *ZEP* is an important mediator of carotenoid accumulation in yellow-fleshed sweet potatoes [37]. The NCED enzyme also plays an important role in downstream aspects of plant carotenoid biosynthesis pathways [23,38]. In apricots, *NCED* expression is responsible for flesh coloration, causing the low levels of β-carotenoid observed in white apricots [39,40]. Here, white-fleshed S19 storage roots were found to exhibit particularly high levels of *NCED3* (*g36276*) expression, particularly during the S4 stage of development, in line with prior research, and suggesting that this gene may be a central driver of the differences in coloration and carotenoid content between the BS and the S19 sweet potato types.

ABA is a key carotenoid-derived phytohormone that is essential in the context of carotenoid metabolism and biosynthesis [23]. By cleaving the (C_11_–C_12_) double bond in 9-cis-violaxanthin and 9′-cis-neoxanthin, NCED generates xanthoxin, which is an ABA precursor, such that NCED catalyzes the initial step necessary for ABA biosynthesis [24]. In the present analysis, the ABA biosynthesis-related genes *ABA2* (*g34785*) and *CYP707A* (*g34128*) were found to exhibit opposing expression patterns in the two analyzed sweet potato types. Specifically, *ABA2* (*g34785*) was significantly upregulated in BS storage roots relative to those from S19 sweet potatoes at the S2 and S4 stages of development, whereas *CYP707A* (*g34128*) exhibited the opposite expression pattern. *ABA2* has previously been reported by González-Guzmán et al. to encode a key enzyme involved in ABA biosynthesis that catalyzes xanothoxin conversion into abscisic aldehyde [41]. In *Capsicum annuum,* Kim et al. determined that ABA hydroxylation mediated by CYP707A was able to promote the degradation of ABA, and thereby reduce the levels of this phytohormone [42]. Consistently, *ABA2* (*g34785*) and *CYP707A* (*g34128*) expression levels were found to be positively and negatively correlated with the ABA levels in BS and S19 samples. Together, these data support roles for *PSY*, *CHYB*, *ZEP*, *NCED3*, *ABA2*, and *CYP707A* as essential regulators of ABA and carotenoid biosynthesis and metabolism in sweet potato storage roots.

Our study showed that the top 20 DEGs encoding TFs that were identified when comparing the S19 and BS storage roots samples were associated with ABA and carotenoid biosynthesis. MYB, HD-ZIP, bZIP, bHLH, and WRKY have previously been identified as critical TFs involved in coordinating carotenoid biosynthesis [43,44]. Specifically, bHLH has been shown to bind the *PSY* promoter and to thereby suppress the expression of this gene, thus reducing carotenoid accumulation [43]. Moreover, bZIP has been reported to influence total carotenoid content in tomatoes [45], while MYB plays a similar role in wolfberries [46]. In line with these prior results, marked bHLH, bZIP, and MYB upregulation was observed in S19 storage root samples relative to those from BS sweet potatoes.

Strikingly, the two sweet potato types exhibited opposite expression patterns for the TF-encoding *FAR1* (*g1323*) and *OFP* (*g10779*) genes. In a prior report, Tang et al. highlighted the importance of FAR1 as a positive regulator of Arabidopsis ABA signaling [47]. Global transcriptional profiling of Arabidopsis specimens further revealed that OFP is a transcriptional repressor capable of controlling ABA signal transduction [48]. We showed that significant increases in *FAR1* (*g1323*) expression were evident in S19 storage roots relative to those from BS sweet potatoes, whereas *OFP* (*g10779*) expression was markedly reduced in S19 samples, as compared to BS samples. This may at least partially account for the observed differences in ABA content in these two sweet potato types [49]. ABA is a key phytohormone that is derived from carotenoid metabolite precursors [23]. These two TFs may thus serve as important regulators of the interplay between carotenoid metabolism and ABA biosynthesis in sweet potato storage roots.

## 5. Conclusions

The mechanisms underlying carotenoid biosynthesis were assessed through comprehensive metabolomic and transcriptomic analyses of the storage roots from two sweet potato types exhibiting different levels of carotenoid content. In this study, *PSY*, *CHYB*, *ZEP*, *NCED3*, *ABA2*, and *CYP707A* were identified as critical genes associated with the observed differences in carotenoid and ABA content in these two sweet potato types. The OFP and FAR1 transcription factors were also identified as important regulators of ABA biosynthesis in sweet potatoes. Carotenoid metabolism is closely related to the biosynthesis of the ABA, and carotenoids are precursors of the ABA. These results enabled the establishment of a proposed integrated network controlling the metabolism and biosynthesis of carotenoids and ABA in sweet potato storage roots. However, further work will be critical to validate these results and to clarify the role that other genes and regulatory mechanisms play in shaping these processes.

## Data Availability

The data presented in the study are deposited in the NCBI SRA database, accession number PRJNA873221.

## Supplementary Materials

The following supporting information can be downloaded at: https://www.mdpi.com/article/10.3390/metabo12111010/s1, Figure S1: Proportions of carotenoid levels and PCA analyses for the indicated samples; Figure S2: KEGG classifications between different samples; Figure S3: KEGG classifications for the comparison of S19-S2 and BS-S2 samples; Figure S4: KEGG classifications for the comparison of the S19-S4 and BS-S4 samples; Table S1: Summary of RNA-seq data and mapped reads for mRNA; Table S2: Statistical results of transcriptome data comparison with reference genomes; Table S3: GO enrichment; Table S4: Statistical table of DEGs in four groups of BS and S19; Table S5: Statistical table of DAMs in four groups of samples; Table S6: TFs classification; Table S7: The gene-specific primers; Table S8: The gene-specific primers.
