# Supplementary material for: Integrated Metabolomic and Transcriptomic Analyses Reveal the Basis for Carotenoid Biosynthesis in Sweet Potato (Ipomoea batatas (L.) Lam.) Storage Roots
Source: Metabolites. 2022 Oct 23;12(11):1010. doi: 10.3390/metabo12111010 (PMC9699360; doi:10.3390/metabo12111010)
Supplement: Supplementary file 1 [file metabolites-12-01010-s001.zip › Supplementary Figure S1. Proportions of carotenoid levels and PCA analyses for the indicated samples..pdf]

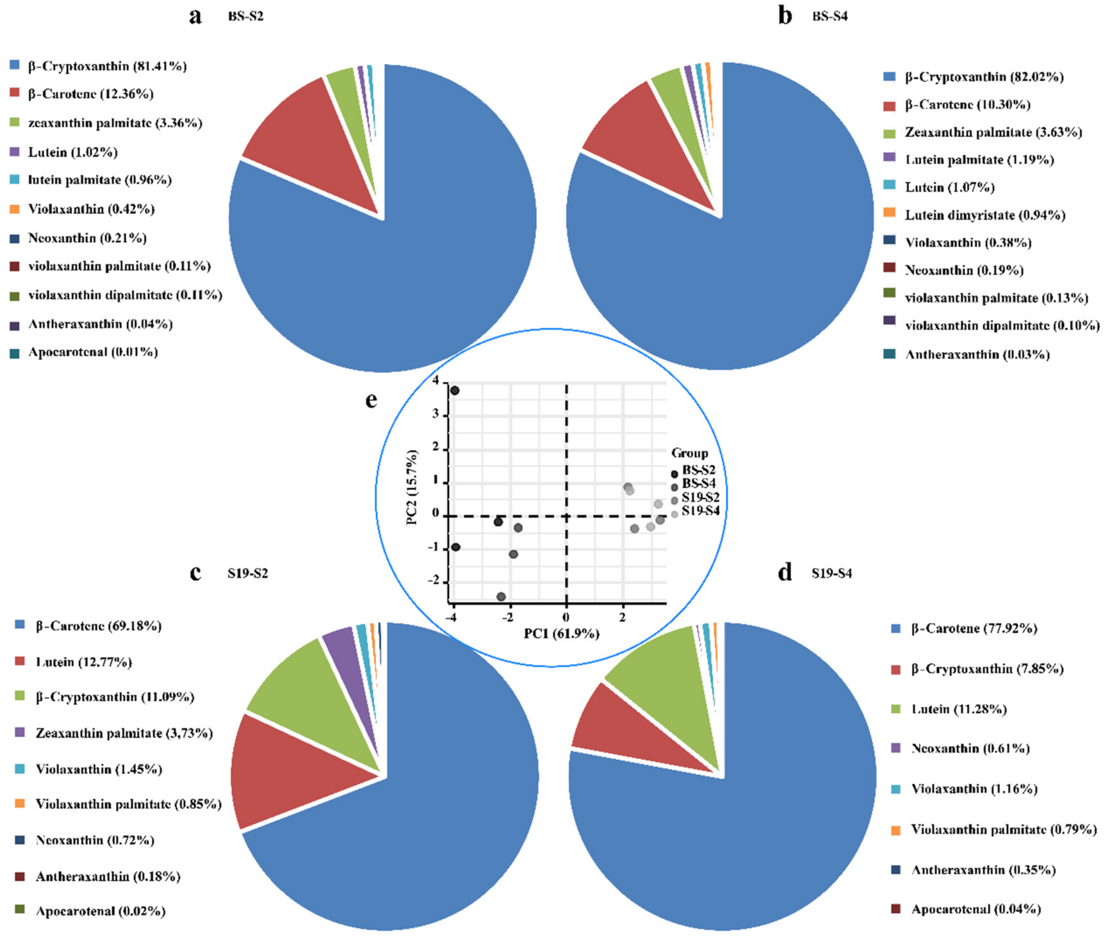

Supplementary Figure S1. Proportions of carotenoid levels and PCA analyses for the indicated samples. (a-d) The proportions of different carotenoids in the indicated sweet potato samples. (e) Principal Component Analysis plots for the indicated metabolites.
