# Supplementary material for: Integrated Metabolomic and Transcriptomic Analyses Reveal the Basis for Carotenoid Biosynthesis in Sweet Potato (Ipomoea batatas (L.) Lam.) Storage Roots
Source: Metabolites. 2022 Oct 23;12(11):1010. doi: 10.3390/metabo12111010 (PMC9699360; doi:10.3390/metabo12111010)
Supplement: Supplementary file 1 [file metabolites-12-01010-s001.zip › Table S1. Summary of RNA-seq data and mapped reads for mRNA. .pdf]

Table S1. Summary of RNA-seq data and mapped reads for mRNA.

Sample	Raw Reads	Clean Reads	Clean Base(G)	Error Rate(%)	Q20(%)	Q30(%)	GC Content(%)
BS-S2-1	47214446	44894540	6.73	0.03	96.84	91.68	48.33
BS-S2-2	47964312	45110380	6.77	0.03	96.79	91.56	48.61
BS-S2-3	48402810	45157956	6.77	0.03	97.00	92.04	49.02
BS-S4-1	45100532	42299524	6.34	0.03	96.43	90.60	48.96
BS-S4-2	45638012	43296616	6.49	0.03	97.03	92.03	48.16
BS-S4-3	42557026	40352728	6.05	0.03	96.90	91.75	48.38
S19-S2-1	47343718	45007686	6.75	0.03	97.06	92.15	48.36
S19-S2-2	46731876	44715466	6.71	0.03	97.02	92.06	48.92
S19-S2-3	46520062	44464158	6.67	0.03	96.86	91.63	47.62
S19-S4-1	47924050	45809036	6.87	0.03	96.91	91.79	46.21
S19-S4-2	46170330	44122566	6.62	0.03	96.75	91.40	45.64
S19-S4-3	44706556	42636324	6.40	0.03	96.86	91.63	47.56
