# Supplementary material for: Integrated Metabolomic and Transcriptomic Analyses Reveal the Basis for Carotenoid Biosynthesis in Sweet Potato (Ipomoea batatas (L.) Lam.) Storage Roots
Source: Metabolites. 2022 Oct 23;12(11):1010. doi: 10.3390/metabo12111010 (PMC9699360; doi:10.3390/metabo12111010)
Supplement: Supplementary file 1 [file metabolites-12-01010-s001.zip › Table S2. Statistical results of transcriptome data comparison with reference genomes. .pdf]

Table S2. Statistical results of transcriptome data comparison with reference genomes.

Sample	Total Reads	Reads mapped	Unique mapped	Multi mapped
BS-S2-1	44894540	38146209(84.97%)	32929203(73.35%)	8802082(11.62%)
BS-S2-2	45110380	38163323(84.60%)	32657175(72.39%)	9480844(12.21%)
BS-S2-3	45157956	38275128(84.76%)	32513513(72.00%)	9744092(12.76%)
BS-S4-1	42299524	35729762(84.47%)	30542153(72.20%)	8744012(12.26%)
BS-S4-2	43296616	36949575(85.34%)	32016651(73.95%)	8302765(11.39%)
BS-S4-3	40352728	34510719(85.52%)	29627814(73.42%)	8253846(12.10%)
S19-S2-1	45007686	38631146(85.83%)	32208681(71.56%)	10815311(14.27%)
S19-S2-2	44715466	38499761(86.10%)	30647969(68.54%)	14153885(17.56%)
S19-S2-3	44464158	37980234(85.42%)	32986455(74.19%)	8632176(11.23%)
S19-S4-1	45809036	39093257(85.34%)	35686835(77.90%)	5580692(7.44%)
S19-S4-2	47686774	41651652(87.34%)	36821145(77.21%)	8265663(10.13%)
S19-S4-3	42636324	36532847(85.68%)	31492576(73.86%)	8440841(11.82%)
