# Supplementary figures and images for: Integrated Metabolomic and Transcriptomic Analyses Reveal the Basis for Carotenoid Biosynthesis in Sweet Potato (Ipomoea batatas (L.) Lam.) Storage Roots
Source: Metabolites. 2022 Oct 23;12(11):1010. doi: 10.3390/metabo12111010 (PMC9699360; doi:10.3390/metabo12111010)

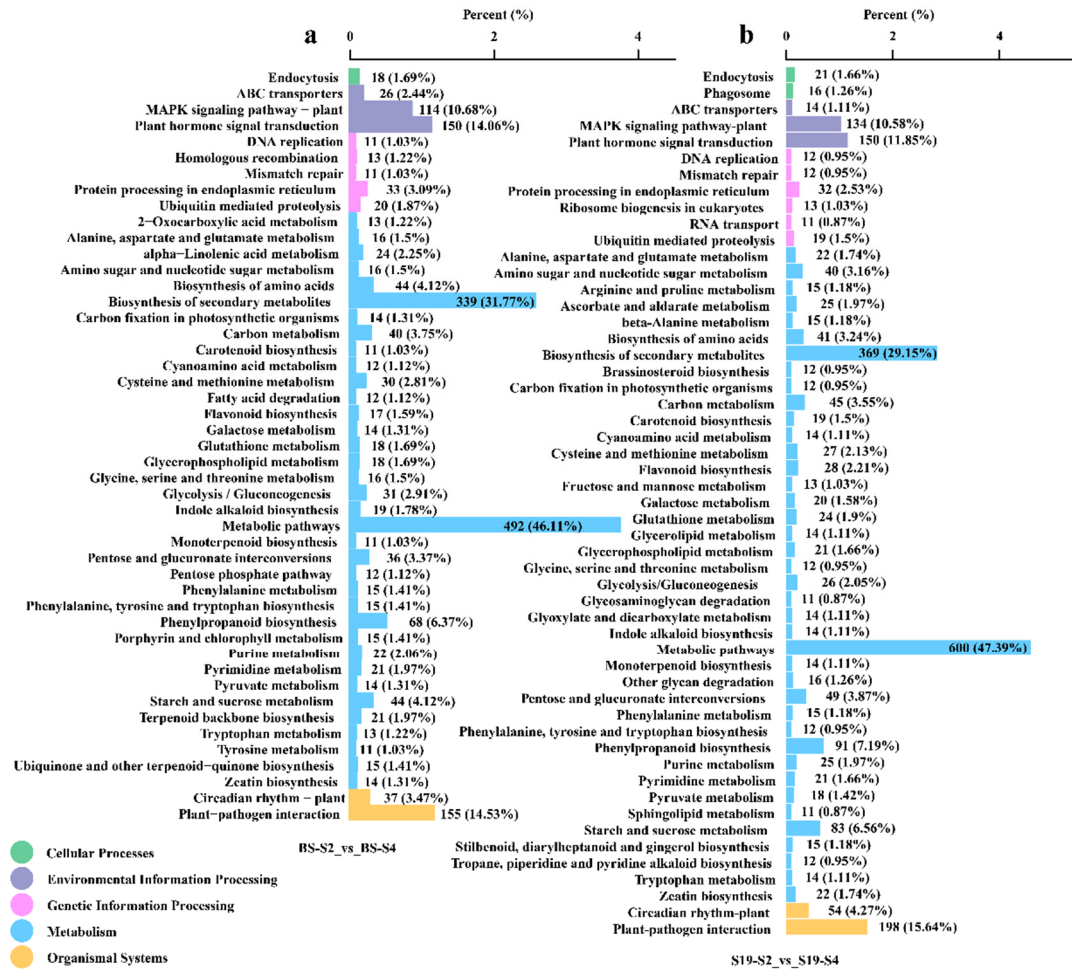

Supplementary Figure S2. KEGG classifications between different samples. (a) BS-S2 vs. BS-S4. (b) S19-S2 vs. S19-S4.

Supplement: Supplementary file 1 [file metabolites-12-01010-s001.zip › Supplementary Figure S2. KEGG classifications between different samples..pdf]

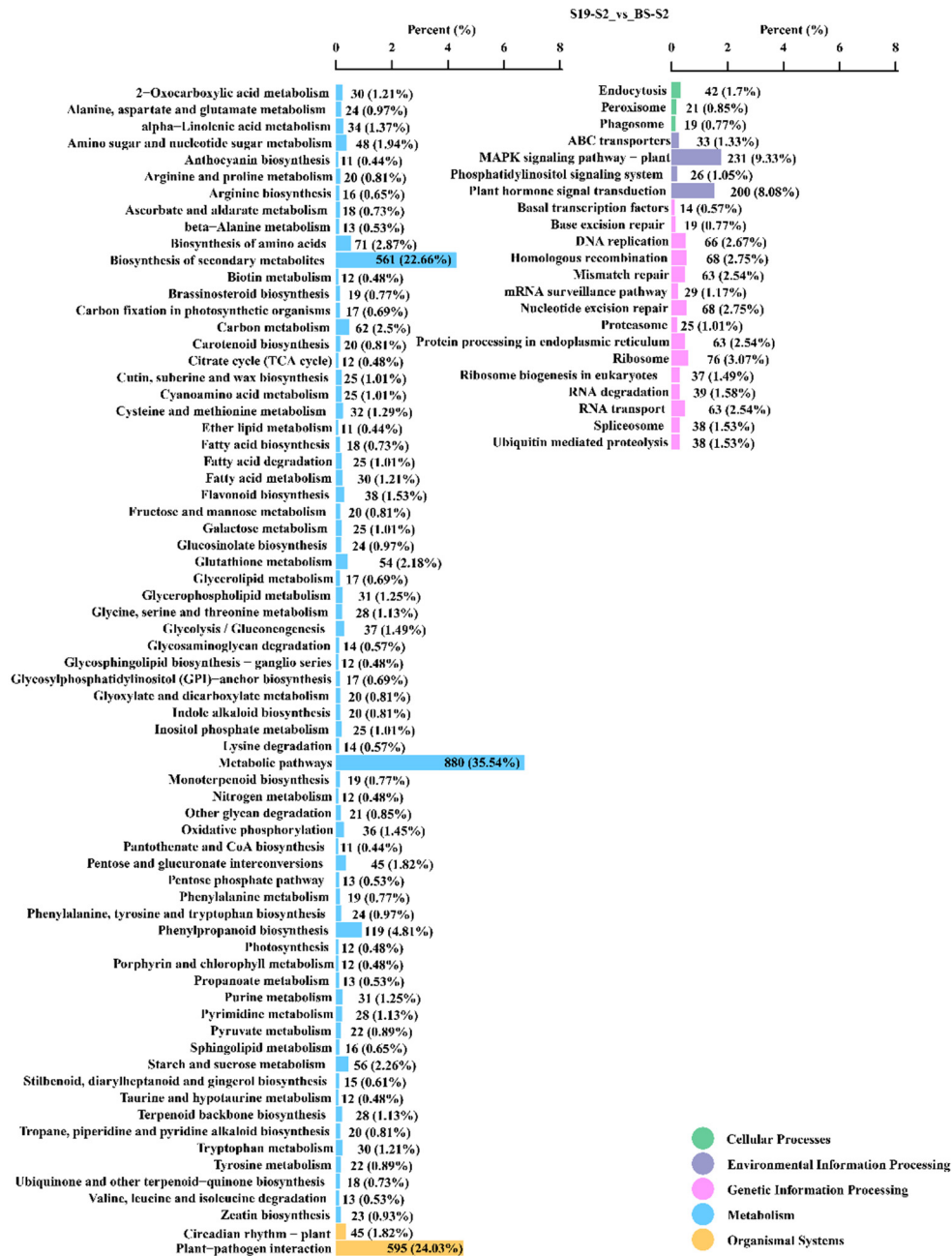

Supplementary Figure S3. KEGG classifications for the comparison of S19-S2 and BS-S2 samples.

Supplement: Supplementary file 1 [file metabolites-12-01010-s001.zip › Supplementary Figure S3. KEGG classifications for the comparison of S19-S2 and BS-S2 samples. .pdf]

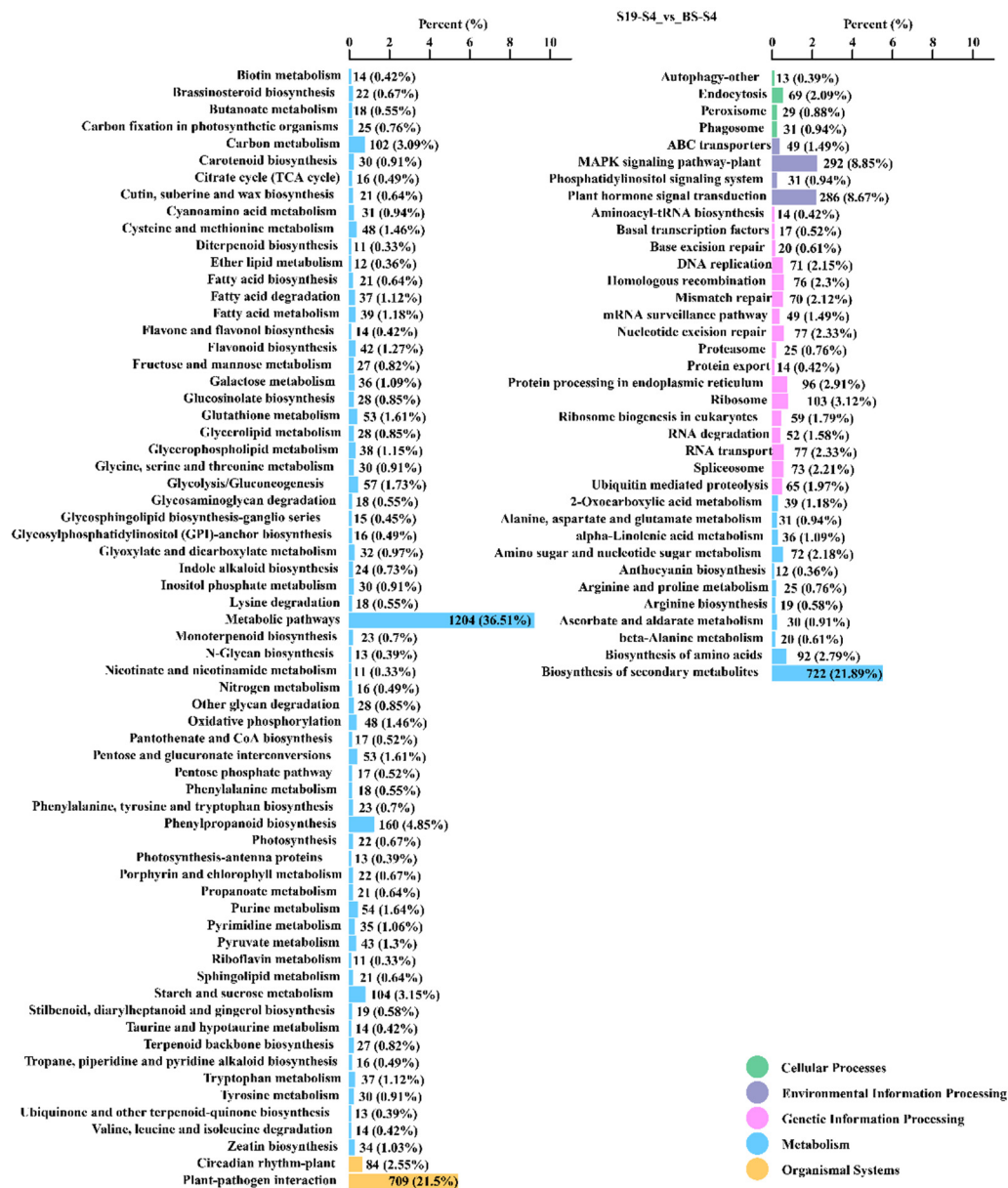

Supplementary Figure S4. KEGG classifications for the comparison of the S19-S4 and BS-S4 samples.

Supplement: Supplementary file 1 [file metabolites-12-01010-s001.zip › Supplementary Figure S4. KEGG classifications for the comparison of the S19-S4 and BS-S4 samples..pdf]
